# Quadriceps Electromyographic Activity in Closed and Open Kinetic-Chain Exercises with Hip-Adductor Co-Contraction in Sedentary Women

**DOI:** 10.3390/ijerph191912929

**Published:** 2022-10-09

**Authors:** Parinyathip Thongduang, Uraiwan Chatchawan, Rungthip Puntumetakul, Junichiro Yamauchi, Punnee Peungsuwan

**Affiliations:** 1 Research Center in Back, Neck, Other Joint Pain and Human Performance (BNOJPH), Khon Kaen University, Khon Kaen 40002, Thailand; 2 School of Physical Therapy, Faculty of Associated Medical Sciences, Khon Kaen University, Khon Kaen 40002, Thailand; 3 Graduate School of Human Health Sciences, Tokyo Metropolitan University, Tokyo 191-0065, Japan

**Keywords:** quadriceps, hip adduction, electromyography, muscle weakness, home-based exercise

## Abstract

Background: Different closed and open kinetic-chain exercises with hip-adductor co-contraction have different effects on quadriceps activity. The aim of this study was to investigate the difference in quadriceps activity during the squat (SQ) and knee extension (KE) and straight leg raise (SLR) exercises with and without hip adduction in sedentary women. Methods: Twenty-eight sedentary women aged 44.5 ± 8.5 years were recruited. They performed three exercises with and without hip adduction. Surface electromyography (sEMG) activity was measured on the rectus femoris (RF), vastus medialis oblique (VMO) and vastus lateralis (VL) muscles. The levels of sEMG activities of the three muscles were compared among the six exercises using a repeated-measures ANOVA. Results: The findings showed that RF activity was lowest during the SQ alone and highest during the SLR exercise (*p* < 0.05 to 0.001). The VMO activity was significantly greater in the SQH than in the five types of exercises (*p* < 0.05 to 0.001), which led to a significant VMO/VL ratio as well. VL activity increased while the squat with hip adduction and knee extension with hip adduction exercise compared with SQ alone. Conclusion: This study indicates that a closed-chain squat with hip co-contraction can produce the VMO and VMO/VL ratio activity, while an open chain of SLR better activates the RF activity. The findings support the understanding of quadriceps activity in different exercises to be an alternative home-based exercise for physical therapy in women facing muscle weakness.

## 1. Introduction

The quadriceps is a major muscle group used for daily activity, including standing, sitting down, stair climbing and walking. Therefore, quadriceps strengthening is key to maintaining a good level of physical activity and helps to prevent falls in elderly individuals [1,2]. A previous study reported a decrease in muscle strength, especially in the lower limbs, in women between the ages of 40 and 50 [3]. Previous studies have described early declines in strength from approximately ages 35 to 50, becoming more apparent at about the age of 50, with more rapid declines above the age of 65 years [4,5,6]. Women exhibit a slightly greater and more rapid decline in muscle strength than men [7]. Associated with such declines are age-related changes in quadriceps surface-electromyography activity [8]. Quadriceps weakness is a risk factor for the development of knee osteoarthritis [9] and appears to be important in the progression of patellofemoral joint disease [10], especially in women [11]. Early intervention for quadriceps strengthening has been recommended for women over the age of 30 years.

Previous studies have reported that squat exercises combined with hip-adductor co-contraction have additive effects on increasing activity of the vastus medialis oblique (VMO) in adults with no knee pain [12], persons with patellofemoral pain [13], and in both subject groups [14], although conflicting results have also been found [15,16]. Although past studies have investigated the effects of different quadriceps exercises involving hip adductor co-contraction of the VMO in adults, concurrent contraction of the rectus femoris (RF) and vastus lateralis (VL) occur to stabilize the knee joint during motion [17]. However, understanding of the activities of all these muscles is needed for planning strengthening programs, especially in adult women who tend to experience declining strength in all muscle fibers of the quadriceps.

Past inconsistent findings concerning the effects of strength training may be due to differences in training design [12,13,16]. In fact, the dynamic muscular properties of closed (squatting) and open kinetic-chain exercises (knee extension and straight leg raise) of the quadriceps differ. In addition, the effects of leg exercise involving hip-adductor co-contraction should be evaluated and compared between different quadriceps exercises [16]. Squatting (SQ), knee extension (KE) and straight leg raise (SLR) are popular conventional strengthening exercises commonly suggested for home-based programs for patients with knee pain and quadriceps weakness [18]. Concurrent hip adduction during quadriceps exercise may also be recommended; however, there is no evidence-based knowledge concerning this in sedentary adult women. The aim of this study was to evaluate, in sedentary adult women, the effects of six quadriceps exercises, with and without hip adductor co-contraction, on rectus femoris (RF), vastus medialis oblique (VMO) and vastus lateralis (VL) and VMO/VL ratio activity. This finding may provide helpful information to promote quadriceps strengthening in individuals with age-related decline.

## 2. Materials and Methods

### 2.1. Subjects

Twenty-eight healthy women adults participated in the present study. The exclusion criteria were knee abnormalities, or pain in the lower extremities within the last six months and/or a history of orthopedic or neuromuscular conditions. Additionally, those subjects who had a quadriceps angle of more than 18 degrees were excluded because a normal quadriceps angle in women is less than 17 degrees [19]. The required sample size was estimated to be at least 24 persons based on a previous study [13] using g-power software (HHU, Düsseldorf, Germany) calculation. All the participants provided written informed consent. The study protocol was approved by the Ethics Committee for Human Research at Khon Kaen University, Thailand (HE 572147).

### 2.2. Experimental Procedure

**Figure 1:**
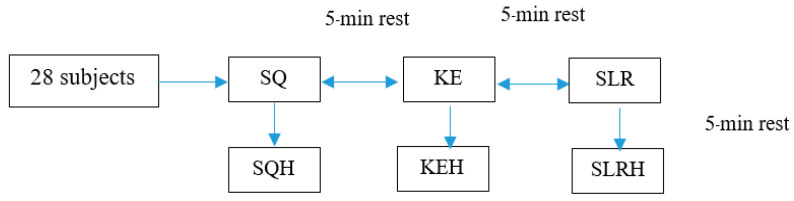
Steps of performing each exercise.

The study was designed to test six different exercises. The trial was designed to compare the quadriceps muscle activities during different leg exercises, including when combined with hip adductor co-contraction. The exercises followed the sequence; (1) squatting (SQ), (2) SQ with hip adduction (SQH), (3) knee extension (KE), (4) KE with hip adduction (KEH), (5) straight leg raising (SLR) and (6) SLR with hip adduction (SLRH) (see Figure 1).

Each participant was asked to practice before performing three tests of each exercise, with a 30 s rest between the trials and a five-minute rest between the exercise conditions (Figure 1). During each leg exercise, surface electromyography (sEMG) was used to determine the superficial quadriceps muscle activity in the rectus femoris (RF), vastus medialis oblique (VMO) and vastus lateralis (VL). The dominant leg of each participant (identified based on the preferred leg) was used for the EMG measurements. A researcher recorded the sEMG and scored the data. Muscle activities were compared between the different leg exercises both with and without hip adductor co-contraction.

### 2.3. Leg-Strengthening Exercises Involving Hip Adductor Co-Contraction

**Figure 2:**
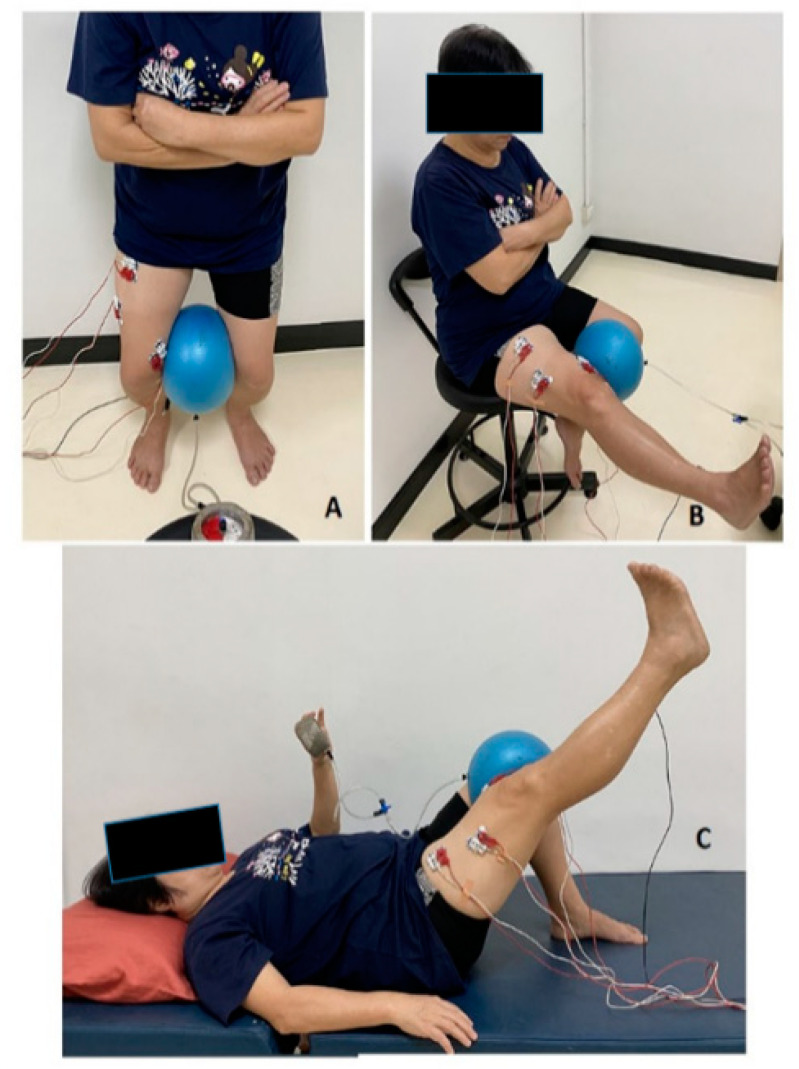
The squat (**A**), knee extension (**B**) and straight leg raise (**C**) exercises with hip adductor co-contraction.

The exercises (SQ, KE and SLR) were all performed with or without hip adduction (Figure 2). During the trials, a metronome was set at 60 bpm to control the speed of the exercises. For the SQ, the participants stood with their backs against a wall and their feet separated at shoulder width and located 10 cm from the wall. The participants then flexed both knees over a four-second eccentric movement time stopping at approximately 60 degrees of knee flexion. The movement range was monitored by a researcher using a goniometer. They then held their position for 3 s (isometric contraction). In this position, the knees were not flexed beyond the toes. The knees were extended back to the standing starting position by the eighth beat within a second. For the KE, the participants sat on a chair. They slowly extended their dominant leg until it was 90 degrees from the initial position and then held isometric contraction in the leg straight for 3 s. For the SLR, the participants started in the supine position with 60 degrees of knee flexion in the non-dominant leg and the dominant leg extended straight. The dominant leg was then raised to 60 degrees of hip flexion, and this isometric contraction was held for 3 s.

For the leg exercises involving hip adduction, the participants maximally squeezed an elastic ball (12 cm in diameter) between their knees and tried to maintain near-maximum hip adduction throughout the process. The maximum hip adduction was determined using a biofeedback pressure gauge. The participants pressed the elastic ball between their knees as hard as possible for 3 s. They performed three trials, with a 30 s rest between the trials and a 5-minute rest after each leg exercise. The three values were averaged to determine the maximum hip adduction.

### 2.4. Electromyographic Analysis

The EMG signals were recorded from the superficial quadriceps muscles (RF, VMO and VL) using a four-channel EMG unit system (MP 100, BIOPAC Systems, Goleta, CA, USA). The sampling rate was 1000 Hz, with a signal amplification of gain ×1000 and a standard mode rejection ratio of 110 dB. The frequency band-pass filter was set between 10 Hz and 500 Hz [18]. The notch filter was set at 50 Hz. Disposable bipolar Ag-AgCl disc surface electrodes (1 cm in diameter) were affixed over the chosen muscle groups, with the inter-electrode distance being approximately 20 mm [14]. The surface EMG electrodes were aligned with the muscle fibers of each muscle individually. The three muscles were palpated to ensure that the electrodes were placed over the bulk of the muscle belly. The assessor confirmed the EMG signals by testing the muscles and evaluating the signals for crosstalk. The RF electrode was placed halfway between the anterior superior iliac spine (ASIS) and the superior part of the patella. The VMO electrode was placed 80% of the way along a line between the ASIS and the joint space in front of the anterior border of the medial collateral ligament. The VL electrode was placed two-thirds of the way along a line from the ASIS to the lateral side of the patella [20]. In addition, a ground electrode was placed over the ipsilateral tibial tubercle of the medial malleolus. The electrodes were connected to an EMG data-collection system, and the signals were collected using customized software (Acqknowledge 3.4 for MP Systems, Biopac Systems Inc., Goleta, CA, USA). The root mean square (RMS) of the EMG signals was used for the analysis. These values were normalized as a percentage of the maximum voluntary isometric contraction (MVIC), which represented the activity of the RF, VMO and VL during each exercise.

The MVIC of activity of each muscle was determined in a sitting position with 90 degrees of hip flexion and 60 degrees of knee flexion. Prior to the MVC test, the participants warmed up their legs by cycling using a stationary bicycle without a load for 5 min. During the maximum knee extension measurement, the participants placed their arms across their chest and kept their hips and trunk on the chair. Verbal encouragement was provided throughout the test to motivate the participants to expend their maximum effort against a resistance above the ankle joint. The participants exerted their maximum isometric force as explosively as possible for ~2 to 3 s in order to retain a force plateau. The participants performed three trials with a 30 s rest period between trials. The average of the three MVIC trials was used for the subsequent calculations [21].

EMG activities were analyzed in portions of a 3 s isometric eccentric holding for the SQ exercise and a 3 s isometric concentric holding for the KE and SLR exercises. A finding provides evidence that isometric muscle contractions loaded in either concentric or eccentric manners elicit similar EMG amplitudes, and are therefore comparable in research settings [22]. The level of difference in EMG activity of the quadriceps femoris was statistically insignificant during eccentric contraction, isometric and concentric contraction [23].

### 2.5. Statistical Analysis

Data analysis was performed using Statistical Package for the SPSS statistical package ( version 18.0, SPSS, Chicago, IL, USA). The data were tested for normality using the Shapiro-Wilk test. A one-way repeated-measures analysis of variance was used to compare the RF, VMO and VL muscle activity differences across six exercise conditions (SQ, SQH, KE, KEH, SLR and SLRH). A Bonferroni post-hoc comparison analysis was performed, and all statistical significance levels were set at *p* < 0.05.

## 3. Results

Twenty-eight healthy women (age: 44.5 ± 8.5 years, range 32–60 years; height: 156.0 ± 4.9 cm; body mass: 53.7 ± 7.2 kg; body mass index: 22.0 ± 2.4 kg/m^2^; mean ± SD) participated the study. The effects of SQ, KE and SLR exercises combining hip adductor co-contraction on the normalized RMS values of quadriceps activity are presented in Table 1. A pairwise comparison of the SQ exercise with or without hip adduction revealed significantly higher level of activity with hip adduction in each muscle type. Such differences were not seen for the KE and SLR exercises.

**Figure 3:**
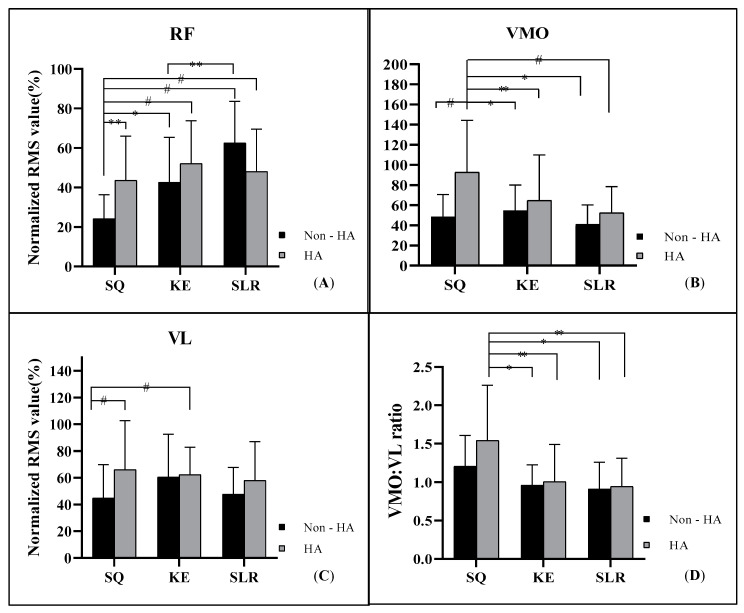
The EMG activity of the RF (**A**), VMO (**B**), VL (**C**) and the VMO/VL ratio (**D**) during the squat (SQ), knee extension (KE), and straight leg raise (SLR) with hip adduction (HA) or without (non-HA). The normalized RMS values are presented as mean ± SD. Significant differences among exercises are indicated by *, *p* < 0.05; **, *p* < 0.001 and ^#^, *p* < 0.001.

Pairwise comparisons of differences among the RF (A), VMO (B), VL (C) activities and the VMO/VL ratio (D) during six types of exercise are shown in Figure 3.

Figure 3A shows that RF activity was significantly lower in the SQ exercise when compared to the SQH (*p* < 0.004), KE (*p =* 0.01), KEH (*p* < 0.001), SLR (*p* < 0.001) and SLRH (*p* < 0.001) exercises. Additionally, the SLR was also greater than the KE (*p* < 0.005). The VMO activity was significantly greater in the SQH than all other exercises, SQ (*p* < 0.001), KE (*p =* 0.04), KEH (*p =* 0.004), SLR (*p* < 0.001) and SLRH (*p =* 0.002), as shown in Figure 3B. Figure 3C shows that the VL activity was significantly greater in the SQH (*p* < 0.001) and KEH (*p* < 0.001) than in the SQ alone. The VMO/VL ratio (Figure 3D) was significantly greater in the SQH exercise than in the KE (*p =* 0.03), KEH (*p =* 0.008), SLR (*p =* 0.02), and SLRH (*p =* 0.006) exercises, but not the SQ.

## 4. Discussion

This study found differences in activities of the RF, VMO, VL and in the VMO/VL ratio between closed and open kinetic-chain quadriceps exercises with and without hip adduction in sedentary women. The maximal VMO and RF activities showed during SQ and SLR exercises, respectively. Concurrent hip adduction with different quadriceps exercises increased the activity of the VMO and VL during the SQH exercise. Likewise, the VMO/VL ratio increased following the performance of the SQH, while there was no significant difference in quadriceps activity in the KEH and SLRH.

The RF activity may be due to numerous nerves firing during the SLR. However, relative to the SLR, the activity of both the VMO and VL increased during the SLRH while that of the RF decreased. Both the KE and KEH exercises caused greater RF activity than the SQ exercise, but this was not the case for the VMO and VMO/VL ratio. The KEH in this study was consistent with a previous demonstration that adding an isometric hip adductor contraction while performing knee extensions did not activate the VMO [24]. The SQ exercise with co-contraction hip adduction produced a superior additive effect on the VMO activity and VMO/VL ratio, which was consistent with previous studies [12,13,25,26]. In contrast, two previous studies found that a dynamic squat with hip adduction did not affect VMO EMG amplitude or VMO/VL ratios [15], and a uniplanar knee extension exercise showed more maximal VMO and VL contractions than isometric knee extension with hip adduction or abduction [16].

Cooperation of the RF, VMO and VL is important in knee-joint function [2]. A possible biomechanical explanation of SLR in the position of hip flexion with knee extension is determined as a longer moment arm of leg position that extends the magnitude of workload. This has the effect of inducing greater RF recruitment, as well as requiring VMO and VL contraction to stabilize the knee in extension. As a result of increased activation of the RF during hip flexion exercises, it has been hypothesized that the SLR exercise would show less EMG activity for the VL and VMO [27,28]. The closed kinetic chain in SQH also affects the overall activation of the VMO and VL. The increases in the VMO and VL activities during the SQH could help to stabilize the knee joint in individuals with patellofemoral pain syndrome [14,29]. Balance of the VMO/VL ratio during the SQH can improve the patellofemoral joint syndrome. This may be of importance in designing training programs aimed toward control of the patellofemoral joint. Previous studies concluded that a closed kinetic-chain exercise promotes more balanced initial quadriceps activation than does exercise in an open kinetic chain [30]. In addition, an increased relative contribution of VMO force produces a reduction in lateral patellofemoral joint loading [31]. The anatomical linkage between the hip adductors and the VMO muscle may partially explain the increased quadriceps activity during the SQH. More specifically, the VMO muscle fibers attach to the tendons of the adductor magnus and adductor longus muscles [32], while the quadriceps muscles are innervated by the femoral nerve and act in concert when extending the knee [33]. Accordingly, the co-contraction of the hip adductors during the squat could increase the recruitment of muscle fibers in both the quadriceps and the gluteus maximus muscles [34]. As a variety of neural and biomechanical mechanisms contribute to the investigated quadriceps exercises, identifying the mechanisms that govern the effects of leg exercises with hip adduction remains a formidable challenge.

Open kinetic-chain or non-bodyweight-bearing exercises [31] and knee extension [16] have a lower or no effect on quadriceps muscle activity when coupled with the co-contraction of hip adductors. The knee joint afferents may affect the controlling neural activity of the quadriceps when combining two different tasks during non-bodyweight-bearing exercises. In addition, no significant differences have previously been found with regard to the level of quadriceps muscle activity between the SLR with or without hip adduction [35]. Similarly, adding hip-adductor co-contraction to the open-chain quadriceps exercises of this study produced no significant increase in the quadriceps muscle activity. However, a previous study suggested that clinicians should re-evaluate their beliefs about open-chain exercises and measure this important variable to improve outcomes for their patients [36].

Closed kinetic-chain exercises have been promoted over open kinetic-chain exercises because the former are more functional, safe and effective for knee rehabilitation [37,38,39]. Clinical trials suggest that closed kinetic-chain programs yield better results in terms of strength [40] and functional [41] performance enhancement. Therefore, quadriceps activity as promoted by the SQH can help to improve joint stability and neural coordination during the hip and knee movements associated with daily living. Our study shows that the SQH using an elastic ball promotes greater RF, VMO and VL activity, while SLR exercise increases RF activity that may be appropriate during rehabilitation in physical therapy clinics and at home for sedentary women.

A limitation of this study was a wide range of participant ages, which might therefore not represent only middle-aged women. However, when participants were split into three age groups (over 30, over 40 and over 50), no difference was found between these when the data were tested separately using a repeated-measures ANOVA. The factors associated with varying physical activity levels among participants were also not considered when evaluating the effects of leg exercises involving hip-adductor co-contraction. In addition, to facilitate the comparison of the physiological effects of the hip-adductor co-contraction during different leg exercises, the loads applied to the muscles should be relatively similar. The body weight of a participant is a load in a closed exercise and obviously varies among individuals. On the other hand, the load applied to the quadriceps muscles during an open-chain exercise varies far less among individuals. Further studies are required to evaluate other exercises and relative exercise loads in older women. Despite these limitations, the findings of this study serve to enhance our understanding of the effects of hip-adductor co-contraction during quadriceps exercises, which should assist with the development of clinic- and home-based rehabilitation exercise programs.

## 5. Conclusions

The activity of the rectus femoris was higher during the straight leg-raise exercise, while that of the vastus medialis oblique was higher in the squat with hip adduction. Two exercise models of squat with hip adduction and straight leg raise should be probably the most chosen among traditional exercises that have a beneficial effect on quadriceps strength for rehabilitation in women older age.

## Figures and Tables

**Table 1:** Comparison of the RMS values of quadriceps EMG activity between exercises with and without hip adduction.

Exercises	Muscles	Non-HA	HA	Mean Difference (95%CI)	*p*-Value
SQ	RF	24.10 ± 12.43	43.66 ± 22.78	19.56 (11.97 to 27.15)	<0.001 ^#^
	VMO	45.28 ± 12.72	84.69 ± 28.61	39.42 (29.36 to 49.48)	<0.001 ^#^
	VL	41.52 ± 18.32	61.79 ± 28.72	20.27 (13.96 to 26.57)	<0.001 ^#^
KE	RF	42.85 ± 23.26	52.22 ± 22.07	9.37 (−0.42 to 19.17)	0.06
	VMO	52.60 ± 23.23	57.96 ± 26.76	5.36 (−4.76 to 15.49)	0.29
	VL	59.60 ± 32.11	61.53 ± 20.11	1.93 (−8.24 to 12.10)	0.70
SLR	RF	61.87 ± 21.25	48.62 ± 21.64	−13.26 (−20.96 to −5.56)	0.41
	VMO	40.00 ± 18.42	52.29 ± 26.52	12.29 (4.78 to 19.81)	0.65
	VL	46.25 ± 19.07	56.27 ± 27.87	10.03 (2.58 to 17.48)	1

The normalized RMS values of the RF, VMO and VL during the six exercises are presented as mean ± SD. RMS, root mean square; SQ *=* squat; KE *=* knee extension; SLR *=* straight leg raise; RF *=* rectus femoris; VMO *=* vastus medialis oblique; VL *=* vastus lateralis. ^#^ indicate *p* < 0.001.

## References

[1] Kostka T., Rahmani A., Berthouze S.E., Lacour J.-R., Bonnefoy M. (2000). Quadriceps muscle function in relation to habitual physical activity and VO2max in men and women aged more than 65 years. J. Gerontol. Ser. A.

[2] Madigan M.L., Lloyd E.M. (2005). Age-related differences in peak joint torques during the support phase of single-step recovery from a forward fall. J. Gerontol. Ser. A.

[3] Borges O. (1989). Isometric and isokinetic knee extension and flexion torque in men and women aged 20–70. Scand. J. Rehabil. Med..

[4] Kallman D.A., Plato C.C., Tobin J.D. (1990). The role of muscle loss in the age-related decline of grip strength: Cross-sectional and longitudinal perspectives. J. Gerontol..

[5] Lynch N.A., Metter E.J., Lindle R.S., Fozard J.L., Tobin J.D., Roy T.A., Fleg J.L., Hurley B.F. (1999). Muscle quality. I. Age-associated differences between arm and leg muscle groups. J. Appl. Physiol..

[6] Lindle R.S., Metter E.J., Lynch N.A., Fleg J.L., Fozard J.L., Tobin J., Roy T.A., Hurley B.F. (1997). Age and gender comparisons of muscle strength in 654 women and men aged 20–93 yr. J. Appl. Physiol..

[7] Doherty T.J. (2001). The influence of aging and sex on skeletal muscle mass and strength. Curr. Opin. Clin. Nutr. Metab. Care.

[8] Hinman R.S., Cowan S.M., Crossley K.M., Bennell K.L. (2005). Age-related changes in electromyographic quadriceps activity during stair descent. J. Orthop. Res..

[9] Øiestad B., Juhl C., Eitzen I., Thorlund J. (2015). Knee extensor muscle weakness is a risk factor for development of knee osteoarthritis. A systematic review and meta-analysis. Osteoarthr. Cartil..

[10] Malek M.M., Mangine R.E. (1981). Patellofemoral pain syndromes: A comprehensive and conservative approach. J. Orthop. Sport. Phys. Ther..

[11] Sheehy P., Burdett R.G., Irrgang J.J., VanSwearingen J. (1998). An electromyographic study of vastus medialis oblique and vastus lateralis activity while ascending and descending steps. J. Orthop. Sport. Phys. Ther..

[12] Earl J., Schmitz R., Arnold B. (2001). Activation of the VMO and VL during dynamic mini-squat exercises with and without isometric hip adduction. J. Electromyogr. Kinesiol..

[13] Wong Y.-M., Straub R.K., Powers C.M. (2013). The VMO:VL activation ratio while squatting with hip adduction is influenced by the choice of recording electrode. J. Electromyogr. Kinesiol..

[14] Miao P., Xu Y., Pan C., Liu H., Wang C.-H. (2015). Vastus medialis oblique and vastus lateralis activity during a double-leg semisquat with or without hip adduction in patients with patellofemoral pain syndrome. BMC Musculoskelet. Disord..

[15] Boling M., Padua D., Blackburn J.T., Petschauer M., Hirth C. (2006). Hip adduction does not affect VMO EMG amplitude or VMO:VL ratios during a dynamic squat exercise. J. Sport Rehabil..

[16] Hertel J., E Earl J., Tsang K.K.W., Miller S.J. (2004). Combining isometric knee extension exercises with hip adduction or abduction does not increase quadriceps EMG activity. Br. J. Sport. Med..

[17] Strazza A., Mengarelli A., Fioretti S., Burattini L., Agostini V., Knaflitz M., Di Nardo F. (2017). Surface-EMG analysis for the quantification of thigh muscle dynamic co-contractions during normal gait. Gait Posture.

[18] Jakobsen T.L., Jakobsen M.D., Andersen L.L., Husted H., Kehlet H., Bandholm T. (2019). Quadriceps muscle activity during commonly used strength training exercises shortly after total knee arthroplasty: Implications for home-based exercise-selection. J. Exp. Orthop..

[19] Emami M., Ghahramani M.-H., Abdinejad F., Namazi H. (2007). Q-angle: An invaluable parameter for evaluation of anterior knee pain. Arch. Iran. Med..

[20] Finni T., Cheng S. (2009). Variability in lateral positioning of surface EMG electrodes. J. Appl. Biomech..

[21] Saiklang P., Puntumetakul R., Selfe J., Yeowell G. (2020). An evaluation of an innovative exercise to relieve chronic low back pain in sedentary workers. Hum. Factors J. Hum. Factors Ergon. Soc..

[22] Garner J.C., Blackburn T., Weimar W., Campbell B. (2008). Comparison of electromyographic activity during eccentrically versus concentrically loaded isometric contractions. J. Electromyogr. Kinesiol..

[23] Seliger V., Dolejš L., Karas V. (1980). A dynamometric comparison of maximum eccentric, concentric, and isometric contractions using EMG and energy expenditure measurements. Eur. J. Appl. Physiol. Occup. Physiol..

[24] Hanten W.P., Schulthies S.S. (1990). Exercise effect on electromyographic activity of the vastus medialis oblique and vastus lateralis muscles. Phys. Ther..

[25] Carlson J., Hobbs L., Smith K. (2010). Isolation of the vastus medialis oblique muscle during semi-squat and straight leg raise exercises. Am. J. Sport. Med..

[26] Raj N.B., Hussin N.A., Bin Simbak N., Safee M.K.M., Rao U.M. (2017). Quadriceps muscle activity during exercise. Res. J. Pharm. Technol..

[27] Livecchi N.M., Armstrong C.W., Cordova M.L., Merrick M.A., Rankin J.M. (2002). Vastus lateralis and vastus medialis obliquus activity during a straight-leg raise and knee extension with lateral hip rotation. J. Sport Rehabil..

[28] Soderberg G.L., Cook T.M. (1983). An electromyographic analysis of quadriceps femoris muscle setting and straight leg raising. Phys. Ther..

[29] Coqueiro K.R.R., Bevilaqua-Grossi D., Bérzin F., Soares A.B., Candolo C., Monteiro-Pedro V. (2005). Analysis on the activation of the VMO and VLL muscles during semisquat exercises with and without hip adduction in individuals with patellofemoral pain syndrome. J. Electromyogr. Kinesiol..

[30] Stensdotter A.-K., Hodges P., Mellor R., Sundelin G., Häger C. (2003). Quadriceps activation in closed and in open kinetic chain exercise. Med. Sci. Sport. Exerc..

[31] Hodges P.W., A Richardson C. (1993). The influence of isometric hip adduction on quadriceps femoris activity. Scand. J. Rehabil. Med..

[32] Bose K., Kanagasuntheram R., Osman M.B.H. (1980). Vastus medialis oblique: An anatomic and physiologic study. Orthopedics.

[33] Thiranagamat R., Lanka S. (1990). Nerve supply of the human vastus medialis muscle. J. Anat..

[34] Sugisaki N., Kurokawa S., Okada J., Kanehisa H. (2014). Difference in the recruitment of hip and knee muscles between back squat and plyometric squat jump. PLoS ONE.

[35] Karst G.M., Jewett P.D. (1993). Electromyographic analysis of exercises proposed for differential activation of medial and lateral quadriceps femoris muscle components. Phys. Ther..

[36] Noehren B., Snyder-Mackler L. (2020). Who’s afraid of the big bad wolf? Open-chain exercises after anterior cruciate ligament reconstruction. J. Orthop. Sport. Phys. Ther..

[37] Rivera J.E. (1994). Open versus closed kinetic chain rehabilitation of the lower extremity: A functional and biomechanical analysis. J. Sport Rehabil..

[38] Snyder-Mackler L. (1996). Scientific rationale and physiological basis for the use of closed kinetic chain exercise in the lower extremity. J. Sport Rehabil..

[39] Lepley L.K. (2015). Deficits in quadriceps strength and patient-oriented outcomes at return to activity after ACL reconstruction: A review of the current literature. Sport. Health.

[40] Augustsson J., Esko A., Thomeé R., Svantesson U. (1998). Weight training of the thigh muscles using closed versus open kinetic chain exercises: A comparison of performance enhancement. J. Orthop. Sport. Phys. Ther..

[41] Witvrouw E., Lysens R., Bellmans J., Peers K., Vanderstraeten G. (2000). Open versus closed kinetic chain exercises for patellofemoral pain: A prospective, randomized study. Am. J. Sport. Med..

